# A Continuous Enzyme-Coupled Assay for Triphosphohydrolase Activity of HIV-1 Restriction Factor SAMHD1

**DOI:** 10.1128/AAC.03903-14

**Published:** 2014-12-23

**Authors:** Laurence H. Arnold, Simone Kunzelmann, Martin R. Webb, Ian A. Taylor

**Affiliations:** a Division of Molecular Structure, MRC National Institute for Medical Research, London, United Kingdom; b Division of Physical Biochemistry, MRC National Institute for Medical Research, London, United Kingdom

## Abstract

The development of deoxynucleoside triphosphate (dNTP)-based drugs requires a quantitative understanding of any inhibition, activation, or hydrolysis by off-target cellular enzymes. SAMHD1 is a regulatory dNTP-triphosphohydrolase that inhibits HIV-1 replication in human myeloid cells. We describe here an enzyme-coupled assay for quantifying the activation, inhibition, and hydrolysis of dNTPs, nucleotide analogues, and nucleotide analogue inhibitors by triphosphohydrolase enzymes. The assay facilitates mechanistic studies of triphosphohydrolase enzymes and the quantification of off-target effects of nucleotide-based antiviral and chemotherapeutic agents.

## INTRODUCTION

Nucleotide analogue inhibitors (Nt-AIs) are a large and important class of molecules employed as antiviral treatments and anticancer therapies. In most instances, the mode of action is through the cellular conversion of a precursor compound to an active triphosphorylated form that might inhibit cellular or viral DNA synthesis, interfere with nucleotide metabolism, or become incorporated as a toxic lesion into the DNA of rapidly dividing tumor cells. SAMHD1 is a recently discovered dGTP-GTP–activated deoxynucleoside triphosphohydrolase (1) that hydrolyzes deoxynucleoside triphosphates (dNTPs) into component nucleosides and an inorganic triphosphate, and SAMHD1 is associated with the downregulation of the cellular dNTP pool (2, 3). Imbalances in dNTP levels have mutagenic and cytotoxic effects that cause genome instability (4) and mitochondrial diseases (5). Moreover, the accumulation of cytosolic nucleic acids from endogenous retroelements is proposed to be a cause of the autoimmunity observed in the SAMHD1-associated disease Aicardi-Goutières syndrome (AGS) (6, 7). In addition, SAMHD1 is an anti-HIV-1 restriction factor that blocks the infection of monocyte-derived dendritic cells (MDDCs) (8), monocyte-derived macrophages (MDMs) (9), and resting T cells (10) through its triphosphohydrolase activity (2). However, although the removal of the SAMHD1 block renders the cells more permissive to infection, the removal also results in greater immune responses (11). These opposing observations implicate SAMHD1 as a target for HIV-1 drug intervention either through the enhancement of SAMHD1 activity to reduce cell susceptibility to infection or through cell-targeted inhibition of SAMHD1 to promote a more robust immune response.

The enzyme has a complex mode of activation and substrate hydrolysis that involves oligomerization linked to an allosteric activation site that requires two nucleotides (12, 13) and an active site that shows exquisite specificity for deoxyribonucleotides over ribonucleotides (1). Given that SAMHD1 both hydrolyzes and is regulated by dNTPs, we developed a continuous assay for SAMHD1 activity in order to determine its quantitative kinetic parameters and to assess if nucleotides, nucleotide analogues, and Nt-AIs are substrates, activators, or inhibitors.

**FIG 1:**
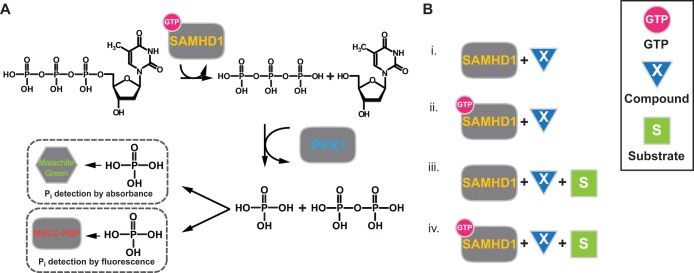
Schematic of the coupled assay for triphosphohydrolase activity. (A) GTP-activated SAMHD1 hydrolyzes TTP, releasing a triphosphate that is cleaved by Ppx1 into pyro- and monophosphates detected by either malachite green (endpoint assay) or MDCC-PBP (continuous assay). (B) The assay was set up for different compound-screening assay questions, as follows. (i) Is the compound both an activator and a substrate? (ii) Is the compound a substrate for GTP-activated SAMHD1? (iii) Is the compound a SAMHD1 activator only? (iv) Is the compound an inhibitor?

The current methods of quantifying SAMHD1 triphosphohydrolase activity are noncontinuous and rely on the analysis of reaction products separated by ion-exchange chromatography (IEX) (1, 14), reverse-phase high-pressure liquid chromatography (HPLC) (12, 27, 28), or thin-layer chromatography (TLC) (17, 18). Therefore, to establish an assay that can be applied in homogeneous and continuous modes, we used a coupled enzyme, the exopolyphosphatase Ppx1 from Saccharomyces cerevisiae, a cytosolic exopolyphosphatase. This enzyme cleaves terminal phosphates processively from a polyphosphate chain and hydrolyzes the triphosphate product of SAMHD1 nucleotide hydrolysis to pyro- and monophosphates. Based on this system, we established two types of assays (shown schematically in Fig. 1A). In endpoint experiments employed for the compound-screening questions (Fig. 1B), released monophosphate was detected with molybdate/malachite green absorbance at 630 nm (19). In continuous assays, phosphate release was monitored in real time to quantify kinetic parameters using the fluorescent phosphate biosensor *N*-(2-[1-maleimidyl]ethyl)-7-(diethylamino)coumarin-3-carboxamide (MDCC)–phosphate-binding protein (PBP) (20, 21).

## MATERIALS AND METHODS

### Protein expression and purification.

The DNA sequence coding for S. cerevisiae exopolyphosphatase 1 (Ppx1) (GenBank accession no. NM_001179332.1) was amplified from genomic DNA by PCR and inserted into a pET-52b expression vector (Novagen) using ligation-independent cloning to produce an amino-terminal StrepII-tag fusion. The insertion sequence was verified by DNA sequencing. The Strep-tagged Ppx1 was expressed upon induction with 1 mM isopropyl-β-d-thiogalactopyranoside (IPTG) in Escherichia coli BL21(DE3) and purified using Strep-Tactin affinity and size-exclusion chromatography on a Superdex 200 into a final buffer of 20 mM Tris-HCl, 150 mM NaCl, 5 mM MgCl_2_, and 2 mM Tris(2-carboxyethyl)phosphine (TCEP) at pH 7.5. The catalytic domain of SAMHD1 (residues 115 to 626) was expressed and purified as previously described (1). The A197C mutation of the E. coli phosphate-binding protein (PBP-A197C) was expressed from vector pET-22b (Novagen) in E. coli strain BL21(DE3) after induction with 1 mM IPTG. Cells were lysed by sonication, purified with PBP-A197C by anion-exchange chromatography, and labeled with *N*-(2-[1-maleimidyl]ethyl)-7-(diethylamino)coumarin-3-carboxamide (MDCC) as previously described (20, 21).

### Ppx1 linked assay-compound screening.

The substrates and compounds were obtained from Jena Biosciences (Jena, Germany) or Thermo Fisher Scientific (Massachusetts, USA). The phosphate concentration was determined using the recommended protocol supplied with the PiColorLock gold phosphate detection system from Innova Biosciences (Cambridge, United Kingdom). Time-course assays were conducted in low-protein-binding 96-well black plates (Corning, USA). The absorbance was measured using a Tecan Safire^2^ (Männedorf, Switzerland) plate reader at 630 nm in a fixed-wavelength mode. All the assays were prepared as master mixes immediately before use in a reaction buffer containing 20 mM Tris-HCl, 150 mM NaCl, 5 mM MgCl_2_, and 2 mM TCEP at pH 7.5.

To determine the triphosphohydrolase activity of SAMHD1, 1 mM substrate and 200 μM allosteric activator were incubated with 5 μM SAMHD1 and 500 nM Ppx1. Fixed-volume samples were removed at predetermined time intervals, and activity was stopped by the addition of 0.25 vol of gold mix (PiColorLock gold) through acidification to a pH of approximately 1.0. After 5 min of incubation at 25°C, 0.1 volume of stabilizer solution (PiColorLock gold) was added, and the mixtures were incubated for a further 30 min at 25°C. The final absorbance was measured as 630 nm by scanning 10 times and averaging data. Each assay was completed in triplicate. The triplicated readings were averaged and normalized by subtracting the average zero-standard optical density. The averaged optical density reading was then compared to a known standard of monophosphate to calculate the concentration of phosphate released.

This protocol was adjusted in the following ways to test the specific screening questions shown in Fig. 1B. To determine whether a compound was both a substrate and an allosteric activator of SAMHD1, only 1 mM of the compound of interest was included in the reaction. To test if a compound was a substrate for activated SAMHD1, 1 mM of the compound plus 200 μM GTP was included. To test if a compound was an allosteric activator, 1 mM TTP substrate plus 200 μM of the compound was employed. For inhibition, 1 mM TTP plus 200 μM GTP plus 200 μM test compound was included in the reaction.

Sample controls were used to check for potential Ppx1 activity on the compounds and the reagents. In these assays, the 200 μM compound was incubated in a reaction buffer at 25°C for 30 s with 500 nM Ppx1, either with or without 200 μM triphosphate. The Ppx1 activity was determined by measuring phosphate release with malachite green detection, as described for the coupled assay above.

### Real-time assay (MDCC-PBP).

The steady-state kinetics of SAMHD1 were determined by a coupled-enzyme assay using Ppx1 and a fluorescent phosphate biosensor, MDCC-PBP. The reactions were set up in 20-μl volumes in 384-well low-protein-binding microplates (Corning, USA), and the time-dependent fluorescence signal was measured using a Tecan Saffire^2^ multiwell plate reader (Männedorf, Switzerland). Solutions containing SAMHD1 (residues 115 to 626), Ppx1, MDCC-PBP, an activator, and an inhibitor (if present) were incubated for 5 min at 25°C before the reaction was initiated by the addition of the substrate. The final concentrations were 100 nM SAMHD1, 10 nM Ppx1, and 40 μM MDCC-PBP. Depending on the experiment, the concentration of the substrate, the activator, or the inhibitor varied while the substrate and/or the activator was held constant at 1 mM and 0.2 mM, respectively. The fluorescence intensity was recorded at 430 nm for excitation and 465 nm for emission wavelengths, with a 7-nm slit width, at 8- to 10-s time intervals over a period of 10 to 30 min. The standard curves were obtained by incubating 40 μM MDCC-PBP with different concentrations of phosphate (P_i_) and measuring the fluorescence, as discussed above. The fluorescence response was linear and in the range of 0 to 20 μM P_i_.

The steady-state rates were obtained from the time courses of P_i_ formation by linear regression of the data points in the linear phase of the reaction. Rates were divided by the SAMHD1 concentration and were plotted versus the activator, substrate, or inhibitor concentration. Apparent dissociation constants for the activator (*K_A_*) or the substrate binding (*K_S_*) and the catalytic constant (*k*_cat_) were determined by nonlinear least-squares fitting using either a hyperbolic or a Hill-function equation in the software package GraFit 7.0.3 (Erithacus Software, United Kingdom) (22). To determine the type of inhibition and the inhibition constant (*K_i_*), experiments were conducted at three constant substrate concentrations (i.e., 1, 0.3, and 0.1 mM TTP), and the inhibitor concentrations varied. Data from the three different experiments were analyzed by a least-squares fit using the equation for competitive inhibition. The fits for the three data sets yielded invariant values for the inhibition constants (*K_i_*), supporting a competitive mode of inhibition. All measurements were performed in triplicate in the reaction buffer.

## RESULTS

### Assessment of Ppx1 activity.

**FIG 2:**
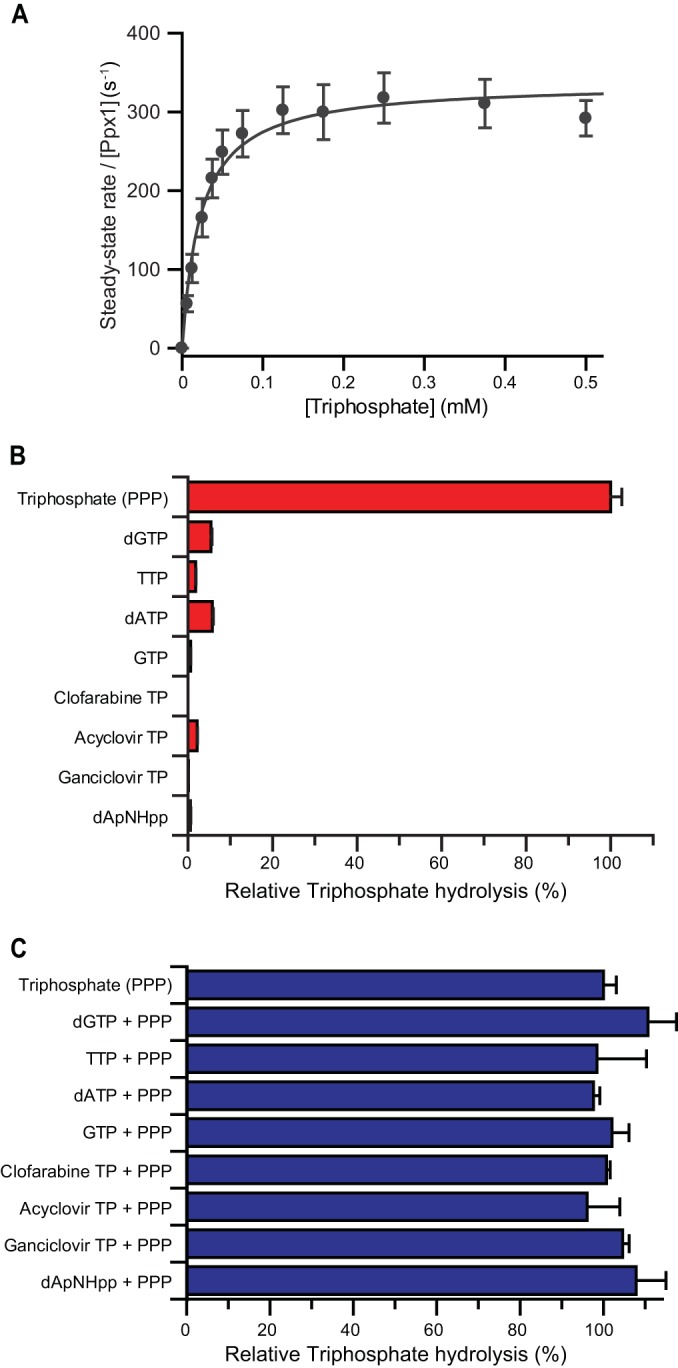
Kinetics and specificity of Ppx1 hydrolysis. (A) Steady-state kinetics of Ppx1-catalyzed triphosphate cleavage to P_i_ and PP_i_ were measured using 40 μM MDCC-PBP, 0.3 nM Ppx1, and various concentrations of the triphosphate. The error bars represent the SEM of three independent measurements recorded at each substrate concentration. Nonlinear least-square fitting to the Michaelis-Menten equation yielded a mean (±SEM) *K_m_* of 23.6 ± 3.2 μM and a mean (±SEM) *k*_cat_ of 338 ± 31 s^−1^. The catalytic constant of Ppx1 triphosphate hydrolysis is at least 200-fold greater than that for SAMHD1-catalyzed nucleotide-triphosphate hydrolysis. (B) Specificity of Ppx1 hydrolysis was measured using the malachite green endpoint assay. The bar chart shows the percentage hydrolysis of nucleoside-triphosphate compounds with respect to the triphosphate control. The error bars represent the SEM of three independent measurements. (C) Inhibition of Ppx1 triphosphate hydrolysis by nucleoside triphosphates. The bar chart shows the percentage hydrolysis of triphosphate in equimolar triphosphate–nucleoside-triphosphate mixtures with respect to a triphosphate-alone control. The error bars represent the SEM of three independent measurements.

The initial control experiments were undertaken to assess the suitability of Ppx1 as a coupled enzyme for the assay. The *K_m_* for Ppx1 hydrolysis of a polyphosphate ranged from 0.004 to 140 μM (23) for P_250_ to P_3_ substrates, with *k*_cat_ values between 180 and 1,500 s^−1^ at 37°C, depending on the preparation (23–25). Quantification of *K_m_* and *k*_cat_ for triphosphate hydrolysis by our recombinant Ppx1 produced in E. coli yielded a *K_m_* of 23.6 μM and a *k*_cat_ of 338 s^−1^ at 25°C (Fig. 2A). Over the triphosphate substrate concentration range employed in the assays, this rate is substantially higher than that of SAMHD1 dNTP hydrolysis (*k*_cat_, ∼1.3 s^−1^) and so is suitable as the coupling enzyme in an assay. The direct Ppx1 hydrolysis of deoxynucleoside triphosphates, or the inhibition of Ppx1 activity by deoxynucleoside triphosphates, was also tested. In these control experiments (Fig. 2B and C), no dNTP hydrolysis by Ppx1 was observable, as reported previously for the ribonucleotides ATP (23) and GTP (25, 26). In addition, no inhibition of Ppx1 hydrolysis of the inorganic triphosphate was apparent when dNTPs were included in the reaction.

### Endpoint assays.

**FIG 3:**
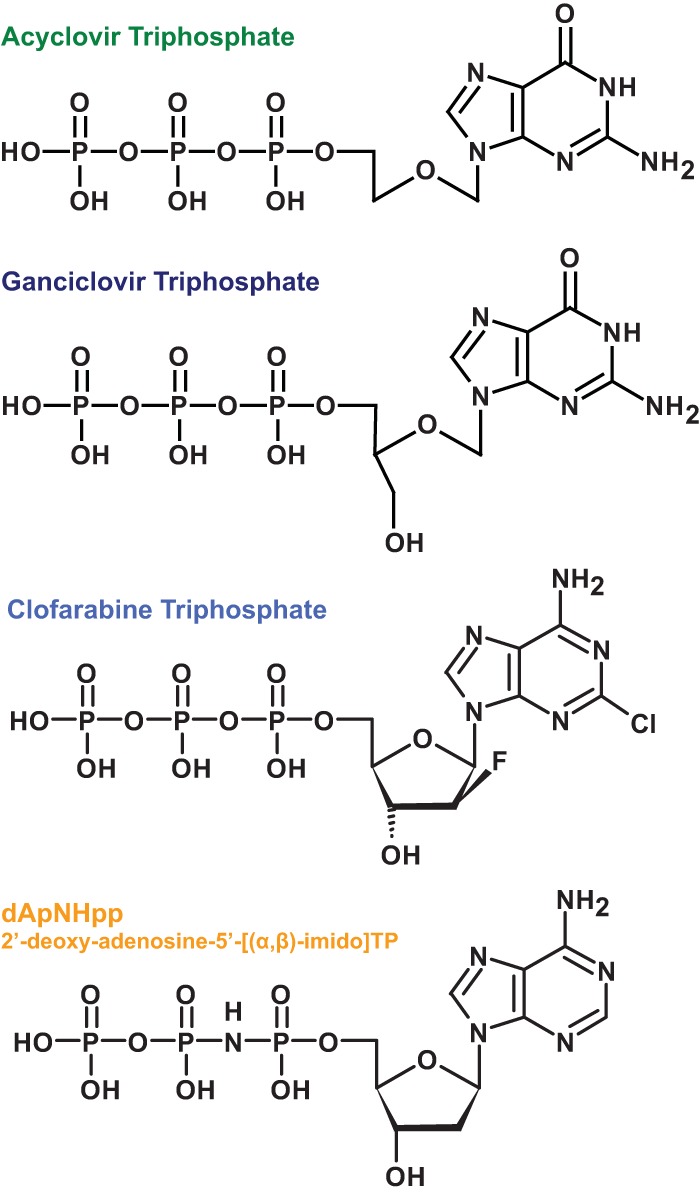
Chemical structures of nucleoside-triphosphate analogues. Chemical structures for the antiviral agents acyclovir-TP and ganciclovir-TP, the anti-leukemia drug clofarabine-TP, and the dATP analogue dApNHpp are shown (from top to bottom, respectively).

To demonstrate the utility of the assay, a selection of deoxyribose and ribose nucleoside triphosphates, together with nucleoside-triphosphate analogues and drug compounds (Fig. 3), were assessed for turnover and/or their capacity to activate or inhibit SAMHD1 activity. The drug molecules selected were the active cellular triphosphate forms of the related antiherpes and anticytomegalovirus compounds acyclovir and ganciclovir (27) and of clofarabine (28), a treatment for acute pediatric lymphoblastic leukemia. In addition, a dATP derivative, dApNHpp, which contains an imido linkage between the α and β phosphates, was also tested.

**FIG 4:**
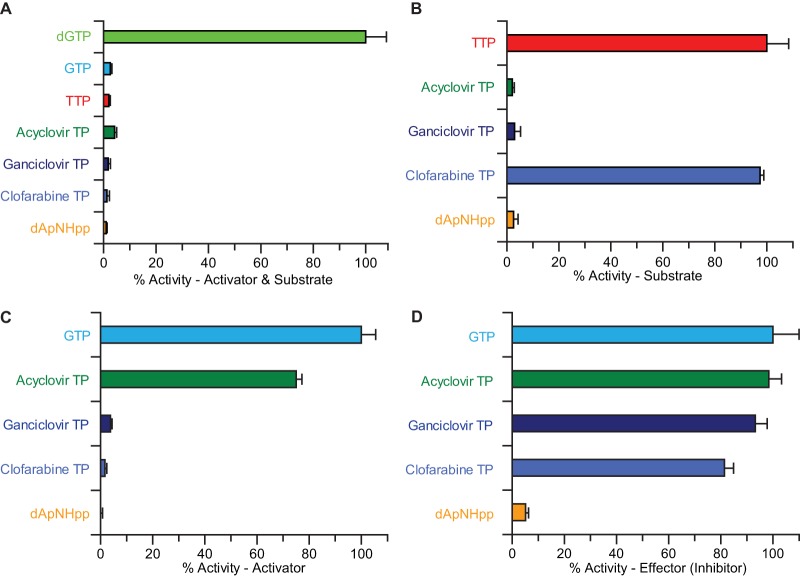
Endpoint assays of SAMHD1 catalysis, activation, and inhibition; results from the malachite green endpoint assays. The bar charts show the percentages of hydrolysis or activity with respect to the control compounds. The error bars represent the standard error of the mean (SEM) of three independent measurements. (A) Assay for compounds that are activators and substrates. The data are expressed relative to that of a dGTP control. (B) Assay for compounds that are hydrolyzed upon inclusion of a SAMHD1 activator (200 μM GTP). The data are expressed relative to that of a control TTP substrate. (C) Assay for SAMHD1 activation. The data are expressed as the percentage of TTP hydrolysis when a test compound was employed as an activator (200 μM) relative to that of the control GTP. (D) Assay for SAMHD1 inhibition. The data are expressed as the percentage of TTP hydrolysis after inclusion of a test compound in addition to the GTP activator at an equimolar concentration (200 μM).

Endpoint assays were used to determine if individual compounds can allosterically activate SAMHD1 and also be hydrolyzed (Fig. 4A). Under these conditions, only dGTP, previously shown to be both a SAMHD1 activator and substrate (1), was hydrolyzed, reaffirming the requirement for a guanine base and the lack of a 2′-hydroxyl for a nucleoside triphosphate to both activate and be hydrolyzed. Although not a substrate, GTP activates SAMHD1 as effectively as dGTP (17); given the abundance of GTP with respect to the cellular dNTP pool, the panel of compounds was also tested to see if they were hydrolyzed by GTP-activated SAMHD1 (Fig. 4B). These data revealed that acyclovir triphosphate (acyclovir-TP), ganciclovir triphosphate (ganciclovir-TP), and dApNHpp were still refractory to hydrolysis but that clofarabine triphosphate (clofarabine-TP) was hydrolyzed at a rate comparable to that of the control TTP.

To ascertain whether any of the compounds can allosterically activate, they were incubated with SAMHD1, and hydrolysis of the TTP was measured (Fig. 4C). Apart from GTP, only acyclovir-TP and ganciclovir-TP contain a guanine base, which is essential for activation at the SAMHD1 allosteric site; as expected, no activation was observed with either clofarabine-TP or dApNHpp. However, whereas acyclovir-TP activated SAMHD1 with around 80% efficiency compared to that of GTP, ganciclovir-TP showed no activation of SAMHD1-TTP hydrolysis, despite being closely related in structure (Fig. 3).

Finally, to assess the inhibition of SAMHD1, each compound was added to a standard reaction of GTP-activated SAMHD1 at a concentration equimolar to the allosteric activator (200 μM), with 1 mM TTP as the substrate (Fig. 4D). Under these conditions, TTP hydrolysis was not significantly inhibited by the addition of acyclovir-TP or ganciclovir-TP and was diminished only slightly by clofarabine-TP. In contrast, the addition of dApNHpp resulted in a substantial reduction in TTP hydrolysis to near background levels, revealing it as a potential inhibitor of SAMHD1 activity.

### Continuous assays.

**FIG 5:**
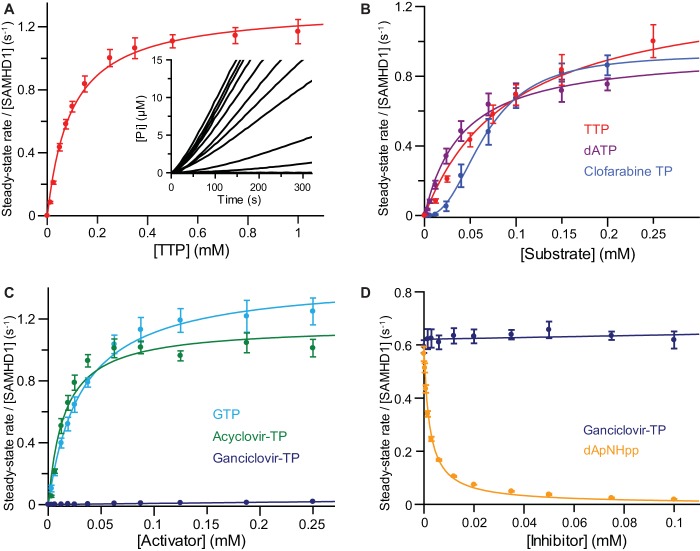
Continuous assays of SAMHD1 catalysis, activation, and inhibition. (A to D) Quantitative continuous assays employing MDCC-PBP readout. The error bars represent the SEM of three independent measurements recorded at each substrate/activator/inhibitor concentration, depending on the assay. (A) Michaelis-Menten analysis of the steady-state kinetics of GTP-activated TTP hydrolysis by SAMHD1. The inset shows the time courses of monophosphate (Pi) formation at different concentrations of TTP from 0 to 1 mM (from bottom to top, respectively). (B) Michaelis-Menten analysis of the steady-state kinetics of GTP-activated SAMHD1 hydrolysis of dATP and clofarabine-TP. The TTP data from panel A are also shown for comparison. (C) Determination of the apparent dissociation constants (*K_A_*) for GTP, acyclovir, and ganciclovir activators. The values are derived from the activator concentration required to support the 50% maximal rate of hydrolysis of a TTP substrate. (D) Determination of inhibition constants (*K_i_*). Data are shown for only the 100-μM TTP substrate with increasing dApNHpp or ganciclovir competitor compounds. Reported *K_i_* values were derived from fitting of data sets employing a substrate over a range of three concentrations.

In order to confirm the findings of endpoint assays and obtain quantitative time-resolved information about the compound hydrolysis, activation, and inhibition of SAMHD1, MDCC-PBP was used to measure phosphate release in real time. The kinetic parameters derived from these assays are summarized in Table 1. A Michaelis-Menten analysis of the steady-state kinetics of TTP hydrolysis by GTP-activated SAMHD1 is shown in Fig. 5A. These continuous measurements (Fig. 5A, inset) were highly reproducible. Moreover, analysis of the data yielded an apparent mean (± standard error of the mean [SEM]) *K_S_* of 96.3 ± 1.7 μM and a mean (±SEM) *k*_cat_ of 1.32 ± 0.07 s^−1^ for TTP hydrolysis, comparable to the quantitative kinetic parameters reported by noncontinuous assays that employed IEX-HPLC (see Fig. S1 in the supplemental material), reverse-phase HPLC, or TLC (13, 15, 16, 18) to measure the product or the substrate. A comparison of the steady-state parameters for GTP-activated SAMHD1 hydrolysis of TTP, dATP, and clofarabine-TP (Fig. 5B) revealed only small differences in the rate of hydrolysis, with the *k*_cat_ for clofarabine-TP reduced from that for TTP but similar to that of the parent purine dATP, although with a significantly sigmoidal concentration dependency (Table 1). SAMHD1 activation by GTP, acyclovir-TP, and ganciclovir-TP was also analyzed by measuring the TTP hydrolysis rate as a function of the activator concentration (Fig. 5C). These data yielded an apparent affinity for an activator molecule (*K_A_*), defined as the concentration of activator required to achieve 50% of the maximal rate of TTP hydrolysis. The *K_A_* values determined in this way were similar for acyclovir-TP and GTP, so even without a deoxyribose moiety but with the guanine, acyclovir-TP still activated SAMHD1 efficiently. In contrast, the structurally related ganciclovir-TP, which also contained a guanine base but maintained a 3′ carbon and hydroxyl, was unable to activate SAMHD1.

**TABLE 1:** Kinetic parameters of SAMHD1 catalysis

Activator	Substrate	Inhibitor	Kinetic parameters^*a*^				
			*K_A_* (μM)	*K_S_* (μM)	*K_i_* (μM)	*k*_cat_ (s^−1^)	*n*_S_
GTP	TTP		32.0 ± 4.2	96.3 ± 1.7		1.32 ± 0.07	
GTP	dATP			42.1 ± 6.9		0.94 ± 0.06	
GTP	Clofarabine-TP			67.6 ± 8.9		0.91 ± 0.07	2.8 ± 0.1
Acyclovir-TP	TTP		15.6 ± 2.2	353 ± 16		1.16 ± 0.05	3.0 ± 0.1
Ganciclovir-TP	TTP		No activation				
GTP	TTP	dApNHpp			1.13 ± 0.07		
GTP	TTP	Ganciclovir-TP			No inhibition		

a Values represent the means ± SEM from experiments performed in triplicate. *K_A_* is the apparent dissociation constant for activator binding, defined as the concentration of the activator required for half-maximal hydrolysis of a TTP substrate. *K_S_* is the apparent dissociation constant for substrate binding, equivalent to the Michaelis constant (*K_m_*) for the combinations of GTP-TTP and GTP-dATP, for GTP–clofarabine-TP, and for acyclovir-TP–TTP. *K_S_* is derived from a Hill-modified Michaelis-Menten model, in which *n*_S_ is the Hill coefficient for substrate binding. *K_i_* is the inhibition constant derived from a steady-state competitive inhibition model. *k*_cat_ is the enzyme turnover derived from the maximum velocity and the total enzyme concentration (*V*_max_/E_0_).

Endpoint assays also revealed an inhibitory effect on SAMHD1 catalysis by dApNHpp, the modified dATP. To quantify this inhibition and to determine if ganciclovir-TP might also have an inhibitory effect, the steady-state hydrolysis of TTP by GTP-activated SAMHD1 was measured in reaction mixtures containing increasing amounts of dApNHpp or ganciclovir-TP. These data (Fig. 5D) clearly showed potent inhibition by dApNHpp but no effect from ganciclovir-TP. Fitting the dApNHpp data with a competitive inhibition model yielded a mean (±SEM) inhibition constant, *K_i_*, of 1.13 ± 0.07 μM, which is 50- to 100-fold lower than the *K_m_* measured for the dNTP substrates.

## DISCUSSION

SAMHD1 is expressed in many immune cells and is a dNTP triphosphohydrolase that is also allosterically activated by nucleoside triphosphates. This has significant implications for the efficacy and off-target effects of Nt-AIs, either by their turnover or through misregulated activation/inhibition of SAMHD1. Therefore, to assess Nt-AI turnover and the modulation of SAMHD1 activity, we developed a highly reproducible assay to screen compound libraries for the effects on SAMHD1 triphosphohydrolase activity that can also be utilized to determine precise *K_m_* and *k*_cat_ values and the inhibition and activation constants for the compounds of interest.

In the endpoint mode, the assay employed large amounts of enzyme combined with malachite green for the detection of the phosphate. This made the assay less sensitive but highly suited to application in compound library screening to assess the capacities of compounds as the substrates, inhibitors, or activators. In the continuous mode, the assay was more time demanding. However, the high sensitivity of the PBP-based detection allowed for an assessment of turnover in the early course of the reaction and so was more amenable to obtaining fully quantitative kinetic parameters once a compound of interest was identified.

Of the sample compounds tested in this study, this assay revealed that the anti-leukemia agent clofarabine-TP is hydrolyzed by SAMHD1 at a rate comparable to that of natural dNTP substrates. The anti-herpes agent acyclovir-TP, while not a substrate, is able to activate SAMHD1 to hydrolyze other dNTPs with efficiency similar to that of GTP. The dATP analogue dApNHpp is a strong competitive inhibitor of SAMHD1 activity. Other *in vitro* studies have demonstrated that SAMHD1 has little or no hydrolytic activity with the anti-HIV Nt-AIs azidothymidine triphosphate (AZT-TP), 2′,3′-didehydro-2′,3′-dideoxythymidine triphosphate (d4T-TP), and 2′,3′-dideoxy-3′-thiacytidine triphosphate (3TC-TP) (14, 29). However, cell-based assays used for investigating the effects of modulating SAMHD1 activity on Nt-AI inhibition of HIV-1 infection have demonstrated that the downregulation of SAMHD1 in monocyte-derived macrophages and activated CD4^+^ T cells results in a large decrease in the efficacy of AZT (14, 29, 30) and, to a lesser extent, in that of other Nt-AIs (29).

It is proposed that the reduction of anti-HIV-1 Nt-AI efficacy results from the increased competition with an expanded dNTP pool for the viral reverse transcriptase active site and from the competition of drug metabolites with nucleotides for the cellular kinase enzymes that direct dNTP synthesis. Therefore, the cellular consequences of Nt-AI hydrolysis or modulation of SAMHD1 activity are far reaching and need to be considered when developing new nucleoside/nucleotide analogues for anticancer and antiviral therapies. Turnover will directly affect the efficacy of an Nt-AI due to the reduction in the number of active drug molecules. Additionally, nonnatural compounds will be delivered into the nucleoside salvage pathway (NSP) with the potential for further off-target secondary effects. The gratuitous activation of SAMHD1 by an Nt-AI will result in enhanced dNTP turnover, starving the cells of essential precursors of DNA and creating a nucleotide deficiency that is associated with DNA damage in replicating cells (31) and the hypermutation of mitochondrial genomes in resting cells (32). The inhibition of SAMHD1 by Nt-AIs may not only raise the level but also alter the balance of the intracellular dNTP pool, as observed in AGS patients (33), resulting in increased DNA damage and a greater susceptibility to viral infection (34, 35). The development of a coupled *in vitro* assay for SAMHD1 triphosphohydrolase activity addresses the need to assess Nt-AIs as SAMHD1 substrates and modulators. Moreover, not only is the quantitative application an essential tool for the evolution and development of Nt-AIs as therapeutic agents, but it also provides insight into the mechanism of SAMHD1 allostery and catalysis.

## Supplementary Material

Supplemental material
