# Supplementary material for: A Continuous Enzyme-Coupled Assay for Triphosphohydrolase Activity of HIV-1 Restriction Factor SAMHD1
Source: Antimicrob Agents Chemother. 2014 Dec 23;59(1):186–92. doi: 10.1128/AAC.03903-14 (PMC4291348; doi:10.1128/AAC.03903-14)
Supplement: Supplemental material [file supp_59_1_186__index.html]

A Continuous Enzyme-Coupled Assay for Triphosphohydrolase Activity of HIV-1 Restriction Factor SAMHD1 — Supplemental material 

# A Continuous Enzyme-Coupled Assay for Triphosphohydrolase Activity of HIV-1 Restriction Factor SAMHD1

## Supplemental material

**Files in this Data Supplement:**

- Supplemental file 1 -

  Description of the IEX-HPLC assay of SAMHD1 triphosphohydrolase activity and a figure showing HPLC traces of 0.2 mM GTP-activated SAMHD1 hydrolysis of 1 mM dATP.

  PDF, 284K
