# Supplementary material for: A Continuous Enzyme-Coupled Assay for Triphosphohydrolase Activity of HIV-1 Restriction Factor SAMHD1
Source: Antimicrob Agents Chemother. 2014 Dec 23;59(1):186–92. doi: 10.1128/AAC.03903-14 (PMC4291348; doi:10.1128/AAC.03903-14)
Supplement: Supplemental material [file AAC.03903-14_zac001153566so1.pdf]

Supplemental Material

A continuous enzyme-coupled assay for triphosphohydrolase activity of HIV-1 restriction factor SAMHD1

Laurence H. Arnold, Simone Kunzelmann, Martin R. Webb and Ian A. Taylor

Supplementary method

IEX-HPLC assay of SAMHD1 Triphosphohydrolase activity

In a typical assay 2-10 μM SAMHD1 was incubated with 0.2 mM GTP activator and 1mM dNTP as substrate in Reaction buffer, 20mM Tris-HCl, 150 mM NaCl, 5 mM $MgCl_2$, 2 mM TCEP (pH 7.5). The reaction was allowed to proceed and samples withdrawn at timed intervals up to 5 minutes and terminated by 10 fold dilution into 18.0% acetonitrile, 25mM Tris-HCl, 1 mM EDTA pH 8.0. The nucleotide hydrolysis reactions were analysed by anion exchange HPLC using a DNA-PAC100 (4 x 50mm) column (Dionex). The column was equilibrated at 30°C at 1 mL/min in 25mM Tris-HCl, 0.5% acetonitrile pH 8.0 (Buffer A). Injected samples (2 nmoles) were eluted with a five-minute isocratic phase of Buffer A followed by linear gradient of 0 to 240 mM NH_4Cl over 12 minutes. Absorbance data from the column eluent was continuously monitored between 200-400nm (2 nm interval) using MD-2010 photodiode array detector (JASCO). Peak integration of the absorbance data recorded at 260 nm was used to quantify the amount of substrate and products during at each time point of the reaction. Initial rates of dNTP hydrolysis were determined from the linear part of plots of substrate against time.

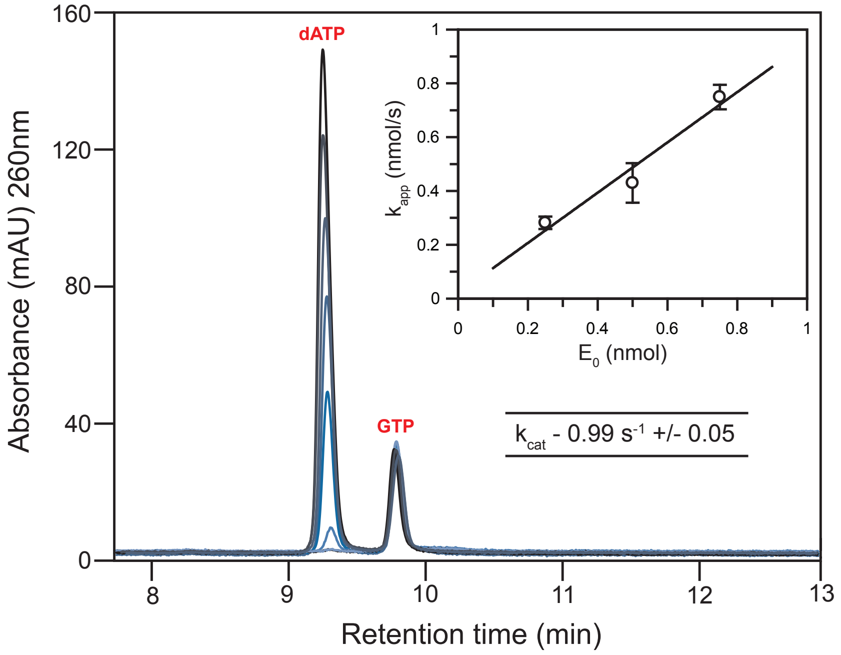

Supplementary Figure S1 - HPLC traces of 0.2 mM GTP activated SAMHD1 hydrolysis of 1 mM dATP. Peaks represent a timecourse of remaining dATP from 0 to 5 minutes at an enzyme concentration of 5 μM. Inset, k_{cat} was determined by linear regression of K_{app} over three enzyme concentrations, 2.5 μM, 5 μM and 7.5 μM. Error bars represent the SEM of three independent measurements recorded at each substrate concentration. See **Table 1** for comparison with k_{cat} determined using the continuous coupled assay.
